# OryzaGP 2021 update: a rice gene and protein dataset for named-entity recognition

**DOI:** 10.5808/gi.21015

**Published:** 2021-09-30

**Authors:** Pierre Larmande, Yusha Liu, Xinzhi Yao, Jingbo Xia

**Affiliations:** 1 DIADE, Univ. Montpellier, IRD, CIRAD, 34394 Montpellier, France; 2 French Institute of Bioinformatics (IFB)—South Green Bioinformatics Platform, Bioversity, CIRAD, INRAE, IRD, Montpellier F-34398, France; 3 Hubei Provincial Key Laboratory of Agricultural Bioinformatics, College of informatics, Huazhong Agricultural University, Wuhan 430070, Hubei Province, China

**Keywords:** biological dataset, gene mention, named entity recognition, natural language processing, *Oryza* species

## Abstract

Due to the rapid evolution of high-throughput technologies, a tremendous amount of data is being produced in the biological domain, which poses a challenging task for information extraction and natural language understanding. Biological named entity recognition (NER) and named entity normalisation (NEN) are two common tasks aiming at identifying and linking biologically important entities such as genes or gene products mentioned in the literature to biological databases. In this paper, we present an updated version of OryzaGP, a gene and protein dataset for rice species created to help natural language processing (NLP) tools in processing NER and NEN tasks. To create the dataset, we selected more than 15,000 abstracts associated with articles previously curated for rice genes. We developed four dictionaries of gene and protein names associated with database identifiers. We used these dictionaries to annotate the dataset. We also annotated the dataset using pre-trained NLP models. Finally, we analysed the annotation results and discussed how to improve OryzaGP.

## Introduction

The past few decades have seen a deluge of information in agronomy. However, a substantial proportion of this information is available in unstructured scientific documents, such as journal articles, reviews, abstracts, and reports. Despite advances in data sciences, innovations in agronomy are still often text-based. One of the challenges is to extract the biological entities and their relationships contained in text fields and scientific papers. Many of these text fields contain molecular mechanisms and phenotypes of interest that are often described by complex expressions associating biological entities linked by specialised semantic relationships (e.g., "*Ehd1 and *Hd3a* can also be down-regulated by the photoperiodic flowering genes Ghd7 and Hd1*" source PMID: 20566706). To address this issue, the objective is to develop computational tools to extract biological entities and their relationships in order to extract relevant information—here, the entities *Ehd1*, *Hd3a*, *Ghd7*, and *Hd1* and the down-regulated relationship. The biomedical field has long experience in developing NLP approaches. The Biocreative [1] and BioNLP conferences [2] have demonstrated numerous advances in this area achieved through the development of datasets and tools. However, little research has been done on this issue in plant science and, more precisely, in the rice sector. For these reasons, we developed a dedicated dataset for rice named OryzaGP. The first release of OryzaGP was initially published in 2019 during BLAH5. The first version originally gathered relatively few PubMed abstracts and focused on named entity recognition (NER) by providing only entities tagged with gene or protein labels. In this new version, we updated the number of PubMed abstracts and provided both NER and the results of named entity normalisation (NEN) when available. Moreover, we tried to merge several database identifiers coming from different resources under the same name. The next section will describe the procedure of building the OryzaGP dataset and how it was annotated.

## Methods

Similarly to the first version, we started by downloading the Oryzabase reference datasets from the Oryzabase [3] web application. Oryzabase provides a manually curated dataset for new rice-related PubMed entries. We filtered out a list of PubMed identifiers that we used to create the OryzaGP_2021 project on PubAnnotation [4]. PubAnnotation [5] is a repository of text annotations related to literature in the life sciences, such as PubMed or PMC articles. It also provides features to create, manage, and access annotations through APIs.

Annotations were conducted through two applications: PubDictionary and HunFlair [6]. PubDictionary is a repository of public dictionaries for the life sciences. It was developed as a model annotation service for PubAnnotation and provides the RESTFul API for dictionary-based text annotation. HunFlair is a NER tagger covering five biomedical entity types. It is integrated into the Flair NLP framework, and it uses a character-level language model pre-trained on roughly 24 million biomedical abstracts and 3 million full texts.

**Fig. 1.**
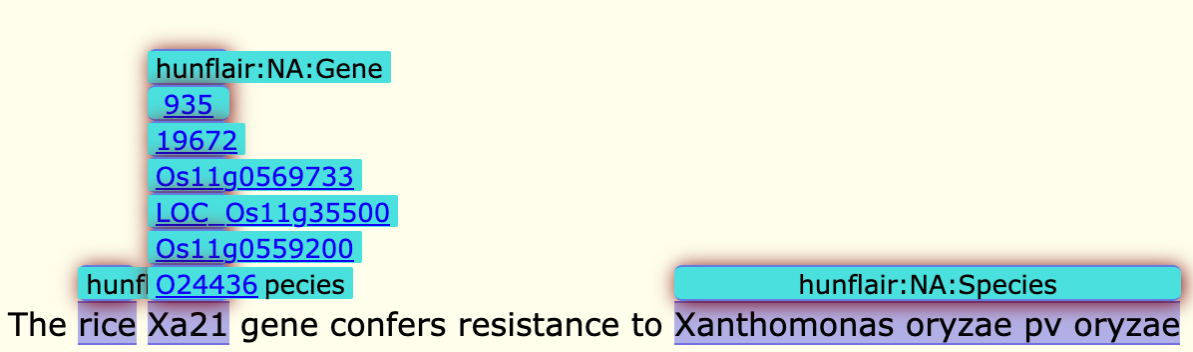
Example of merged annotations with TextAE tool.

In order to use PubDictionary to annotate OryzaGP, we created several dictionaries of gene/protein entities. We first downloaded the Oryzabase gene dataset, which contains several gene mentions associated with database identifiers. We created the Oryzabase dictionary containing labels, gene names, symbols, synonyms and Oryzabase identifier URIs. Next, we repeated the same process to create the RAPDB [7], MSU [8], and UniProt [9] dictionaries. Additionally, we refined the RAPDB and UniProt dictionaries by adding new entries extracted from the RAPDB gene datasets. All these dictionaries were uploaded to PubDictionary and used to create several annotators. Table 1 shows the size (i.e., the number of entries) of these dictionaries. Finally, we utilized PubAnnotation to run several annotations on OryzaGP using these dictionaries. We merged these annotations in a single project (Fig. 1).

HunFair, which comes with models for genes, proteins, chemicals, diseases, species and cell lines, is an advanced NER tagger for biomedical texts. Compared with other biomedical NER tools, such as GNormPlus [10] and HUNER [11], HunFlair showed better performance on the BioNLP 2013 CG [12] and Plant-Disease corpus [13]. In the OryzaGP project, we imported the HunFlair pre-trained model directly to annotate the abstracts in OryzaGP. HunFlair annotated each abstract with genes, proteins, chemicals, diseases, and species, and converted the JSON results into a format that met the requirements of the PubAnnotation platform. All annotations created by HunFlair were prefixed with *hunflair:NA* plus the entity type (e.g., gene, disease, cell line, chemical, and species).

## Results

Compared to the first version of OryzaGP, this updated version was significantly improved. Table 2 compares basic statistics on both versions. The number of articles was increased from 10,000 to 15,000, and consequently the number of sentences and words increased as well. The number of annotations also increased. In the first version, the annotations were produced with an improved Bi-LSTM-CRF model from [14,15] previous research [12,13]. Around 29,000 annotations were found. In this current version, we used multiple annotators to achieve this goal (see the Materials and Methods section) and obtained about 1 million annotations (Table 3). The annotations were merged into the single project. Fig. 1 shows an example of merged annotations with the TextAE editor from PubAnnotation. We can see tagged entities with a class label and other entities tagged with database identifiers (i.e., NEN). We obtained NEN results in 64% of cases, which means that nearly two-thirds of the annotations are linked with a database identifier.

To our knowledge, OryzaGP is the first dataset created for genes and proteins in rice species. It can help to better train NLP tools to recognize rice-related biological entities. Moreover, this new version contains a large number of normalized genes and proteins. However, manual checking of these annotations revealed some false positives. For this iteration of the project, it was not possible to develop a strategy to automatically evaluate the rate of false-positive and false-negative annotations. This remains a task for future work.

### Future work

Our future work will first focus on identifying false positives and negatives to improve annotations. We manually observed that false positives often occurred with gene and protein full names. Some annotations did not match the whole sequence of words. Our hypothesis is that there often exist co-occurrences of full names and symbols in the same sentence or abstract. Thus, we will analyze and classify these co-occurrences.

Next, we plan to normalise the annotations done by HunFlair. Some are already merged with NEN, but some are not. We plan to analyse these annotations, especially those standing for gene symbols, and set up a strategy to normalise them.

Finally, we are interested in adding new annotation types such as plant organs or plant traits. Thus, we will create dictionaries and train NLP tools to achieve this goal.

## Figures and Tables

**Table 1.** Description of the dictionaries

Name	Size
OryzaGeneName_Oryzabase	175,158
OryzaGeneName_RAPDB	110,539
OryzaGeneName_MSU	112,309
OryzaGeneName_UniProt	66,934

**Table 2.** Description of the dataset

Name	OryzaGP	OryzaGP 2021
Text genre	Article	Article
Text type	Abstract & Title	Abstract & Title
Entity type	Gene, Protein	Gene, Protein
No. of articles	10,400	15,041
No. of sentences	75,096	150,604
No. of words	2,697,726	4,101,648
No. of annotations	29,098	1,064,353
No. of gene mentions	None	677,938

The number of annotations corresponds to the total annotations detailed in Table 3. The number of gene mentions was calculated from the fourth PubAnnotation (oryzabase.gene, rapdb.gene, uniprot, msu.gene) results because the corresponding dictionaries contained the URIs of the entities.

**Table 3.** Description of the annotations

Annotation type	No. of annotations
hunflair:NA:CellLine	5,195
hunflair:NA:Chemical	86,770
hunflair:NA:Disease	12,369
hunflair:NA:Gene	171,761
hunflair:NA:Species	110,320
PubAnnotation.oryzabase.gene	189,081
PubAnnotation.rapdb.gene	175,285
PubAnnotation.uniprot	140,354
PubAnnotation.msu.gene	173,218
Total	1,064,353
